# Protein aggregation and therapeutic strategies in SOD1- and TDP-43- linked ALS

**DOI:** 10.3389/fmolb.2024.1383453

**Published:** 2024-05-24

**Authors:** Maria Tsekrekou, Maria Giannakou, Katerina Papanikolopoulou, Georgios Skretas

**Affiliations:** ^1^ Institute of Chemical Biology, National Hellenic Research Foundation, Athens, Greece; ^2^ Department of Biology, National and Kapodistrian University of Athens, Athens, Greece; ^3^ Institute for Fundamental Biomedical Research, Biomedical Sciences Research Centre “Alexander Fleming”, Vari, Greece; ^4^ ResQ Biotech, Patras Science Park, Rio, Greece; ^5^ Institute for Bio-innovation, Biomedical Sciences Research Centre “Alexander Fleming”, Vari, Greece

**Keywords:** amyotrophic lateral sclerosis, SOD1, TDP-43, protein misfolding, protein aggregation, neurotoxicity, therapeutics

## Abstract

Amyotrophic lateral sclerosis (ALS) is a fatal neurodegenerative disease with severe socio-economic impact. A hallmark of ALS pathology is the presence of aberrant cytoplasmic inclusions composed of misfolded and aggregated proteins, including both wild-type and mutant forms. This review highlights the critical role of misfolded protein species in ALS pathogenesis, particularly focusing on Cu/Zn superoxide dismutase (SOD1) and TAR DNA-binding protein 43 (TDP-43), and emphasizes the urgent need for innovative therapeutic strategies targeting these misfolded proteins directly. Despite significant advancements in understanding ALS mechanisms, the disease remains incurable, with current treatments offering limited clinical benefits. Through a comprehensive analysis, the review focuses on the direct modulation of the misfolded proteins and presents recent discoveries in small molecules and peptides that inhibit SOD1 and TDP-43 aggregation, underscoring their potential as effective treatments to modify disease progression and improve clinical outcomes.

## Introduction

Precise activity of cellular protein networks is essential for the proper function and fitness of cells and organisms. Protein function highly depends on its three-dimensional structure, i.e., its folding state (Yadav et al., 2019). To ensure functionality, biological systems have evolved protein quality control mechanisms comprising molecular chaperones, proteases and regulatory factors, which supervise protein folding and direct protein species which fail to meet cellular quality control criteria for degradation. Despite the existence of highly specialized protein folding surveillance mechanisms, protein misfolding and aggregation are hallmarks of numerous human diseases (Chiti and Dobson, 2006; Chiti and Dobson, 2017). These diseases range from systemic amyloidoses, where amyloid fibrils accumulate throughout the human body, to neurodegenerative disorders such as Alzheimer’s disease (AD), Parkinson’s disease (PD) and ALS, where protein aggregates are mainly deposited either extracellularly (AD) or intracellularly (PD, ALS) within the central nervous system (CNS).

ALS is a neurodegenerative disease (Hardiman et al., 2017) characterized by the selective degeneration of both upper motor neurons in the motor cortex and lower motor neurons in the brainstem and spinal cord (Tandan and Bradley, 1985; Hardiman et al., 2017; van Es et al., 2017). It is the most common form of motor neuron disease with adult onset and the third most common neurodegenerative disease (Hou et al., 2019). The survival rate is highly variable, with a median rate of about 3–5 years after symptom onset; however, up to 10% of ALS patients survive for more than 10 years (Chio et al., 2009; van Es et al., 2017; Longinetti and Fang, 2019). ALS affects approximately two to four people per 100,000 individuals per year in Caucasian populations and about one person or less per 100,000 individuals annually in Asian and Hispanic populations (Cronin et al., 2007; Al-Chalabi and Hardiman, 2013; Hardiman et al., 2017; Longinetti and Fang, 2019), with the exception of Japan where the incidence rate is closer to the that of Western countries (Doi et al., 2014). Recent studies indicate a trend of rising incidence rates for ALS (Collaborators, 2019; Longinetti and Fang, 2019; Ryan et al., 2019).

ALS has traditionally been classified as either sporadic (sALS), representing approximately 90%–95% of cases, or familial (fALS), typically characterized by Mendelian autosomal dominant inheritance (van Es et al., 2017; Ranganathan et al., 2020; Kirola et al., 2022). The term “sporadic” is applied to cases which are unrelated to family incidence, even when gene mutations associated with or causing ALS are present (Mejzini et al., 2019). Comprehensive analyses from large-scale genome-wide association studies (GWAS) on sALS patients have revealed that the genetic landscape of ALS predominantly comprises rare variants (van Rheenen et al., 2016; van Rheenen et al., 2021). Consequently, the field is progressing toward a more precise genetic classification, emphasizing the identification of risk genes (Cooper-Knock et al., 2021; Brenner and Freischmidt, 2022). Regardless of the subclassification criteria, both forms of ALS are clinically indistinguishable (Couratier et al., 2021), differing only in the age of onset, with fALS occurring about a decade earlier than sALS (Wijesekera and Leigh, 2009).

Familial cases of ALS have facilitated the identification of the involvement of the genetic background in disease pathogenesis (Gros-louis et al., 2006). Currently more than 40 genes have been associated with fALS (van Rheenen et al., 2021; Brenner and Freischmidt, 2022; Suzuki et al., 2023), enabling the generation of animal models carrying ALS-related gene mutations (Bonifacino et al., 2021; Zhu et al., 2023). *In vivo* modelling of ALS has provided a valuable tool to elucidate the pathogenic mechanisms contributing to disease onset and progression, and allowing for targeted drug development (Hardiman et al., 2017; Bonifacino et al., 2021; Zhu et al., 2023). Importantly, research of ALS animal models has revealed an intertwining network of molecular (e.g., aberrant RNA metabolism, impaired protein homeostasis, oxidative stress, mitochondrial dysfunction, impaired DNA repair and dysregulated vesicle transport) and cellular disruptions (such as hyperexcitability, glial dysfunction and axonopathy) that build up to systemic aberrations, ultimately leading to the disease (Hardiman et al., 2017).

ALS is a complex disease, characterized by significant clinical heterogeneity. This heterogeneity is evident in the variability of the site and age of disease onset, the rate of progression, and the degree of cognitive impairment (Chio et al., 2011; Ferrari et al., 2011; Statland et al., 2015). Despite this clinical variability, a cellular hallmark of ALS is the presence of ubiquitinated skein-like or dense and round cytoplasmic inclusions of certain proteins, such as SOD1, TDP-43, and fused in sarcoma (FUS) in motor neurons (Leigh et al., 1988; Lowe et al., 1988). Approximately 97% of ALS cases exhibit TDP-43-positive inclusions (Mackenzie et al., 2007). Notably, TDP-43 cytoplasmic inclusions are also found in other neurodegenerative disorders including Frontotemporal lobar degeneration (FTLD), combined ALS-FTLD, AD and atypical Parkinsonism (de Boer et al., 2020). TDP-43 inclusions are absent in ALS cases caused by mutations in *SOD1* and *FUS*; in these instances, protein deposits consist of the respective mutated gene products (Neumann et al., 2009; Vance et al., 2009).

Considering that aberrant cytoplasmic inclusions are a ubiquitous finding in ALS patients, numerous research efforts have been directed toward inhibiting the aggregation of ALS-related proteins. In this review, we focus on two extensively studied ALS-related proteins associated with protein misfolding and aggregation, SOD1 and TDP-43. We examine the contribution of their accumulation to disease phenotypes and review the recent progress in identifying inhibitors of their aggregation, which could serve as lead molecules for the development of effective anti-ALS treatments.

## Misfolding- and aggregation-mediated toxicity in ALS

### Aggregation-prone proteins in ALS

SOD1 plays a major role in regulating redox potential by catalyzing the conversion of the superoxide anion O_2_^−^ into hydrogen peroxide, which is further processed by other enzymes (Bunton-Stasyshyn et al., 2015; Huai and Zhang, 2019). In its functional form, SOD1 forms a homodimer, with each subunit containing a copper and a zinc ion at its [Cu/Zn] site and an intra-subunit C57-C146 disulfide bond (Valentine et al., 2005; Huai and Zhang, 2019). The discovery of *SOD1* mutations causing fALS in 1993 was a milestone for ALS research (Rosen et al., 1993). To date, more than 200 mutations in human *SOD1* have been associated with ALS (van der Spek et al., 2019). Mutations in *SOD1*, lead to classical dominantly inherited ALS, accounting for about 10%–15% of fALS cases and approximately 1.5% of sALS cases (Gros-louis et al., 2006). Although SOD1 misfolding and aggregation are considered hallmarks of SOD1-fALS (Saccon et al., 2013), SOD1-immunoreactive inclusions have also been detected in a significant percentage of sALS cases (Bosco et al., 2010; Forsberg et al., 2019). As the longest-studied ALS-related protein, SOD1 has been associated with a large number of pathophysiological mechanisms causing neurotoxicity, including protein misfolding, excitotoxicity, oxidative stress, impaired axonal transport, inflammation and mitochondrial dysfunction (Ferraiuolo et al., 2011).

Wild-type SOD1 is an unusually stable protein, which remains active even under denaturing conditions (Arnesano et al., 2004). It is able to maintain its disulfide status inside the reducing cytosolic environment and is highly resistant to proteolysis (Arnesano et al., 2004; Valentine et al., 2005). This remarkable stability is regulated by the disulfide bond and the metalation status, which mutually affect each other (Arnesano et al., 2004; Tiwari et al., 2005; Nordlund et al., 2009). Disruption of the disulfide bond or loss of the metal co-factors can lead to pathogenic misfolding. Specific post-translational modifications (PTMs), including phosphorylation, acetylation, and succinylation, play crucial roles in regulating the structure and functions of SOD1, such as ROS scavenging, cytoskeletal organization, and transcriptional activity (Banks and Andersen, 2019), while others like sumoylation and oxidation can lead to misfolding and aggregation (Fei et al., 2006; Xu et al., 2018; Trist et al., 2022).

Interestingly, mutations associated with ALS have been identified across all five exons of human SOD1. These mutations impact the structure of SOD1 in different ways, often leading to varying degrees of misfolding, aggregation and consequent variable toxicities (Brasil et al., 2018). Initially, it was proposed that ALS-causing mutations in SOD1 were linked to a loss of enzymatic function (Rosen et al., 1993; Saccon et al., 2013); however, subsequent studies in SOD1 mice contradicted this notion (Gurney et al., 1994; Reaume et al., 1996). Mutant SOD1 retaining partial or complete enzymic function was found to induce ALS‐like phenotypes (Gurney et al., 1994), and *Sod1* knockout mice did not develop ALS (Reaume et al., 1996). In contrast, ALS-related SOD1 variants are associated with decreased metal binding, reduced formation of a stabilizing intramolecular disulfide bond, diminished structural stability and an increased tendency to monomerize and aggregate (Ray et al., 2004). Importantly, the aggregation propensity of several SOD1 variants has been linked to life expectancy after the onset of ALS symptoms in both humans and transgenic mouse models (Wang et al., 2008; Prudencio et al., 2009; Pratt et al., 2014; Lang et al., 2015; McAlary et al., 2020).

TDP-43, encoded by the *TARDBP* gene, was identified in 2006 as a major component of cytoplasmic protein inclusions accompanied by nuclear clearance of the protein, as observed in motor neurons of ALS cases (Arai et al., 2006; Neumann et al., 2006; Mackenzie et al., 2007). TDP-43 pathology is present in nearly all ALS cases except for those caused by mutations in *SOD1* or *FUS*. *TARDBP* mutations account for approximately 5% of familial and almost 1% of sporadic ALS cases. It is an RNA-binding protein (RBP) involved in the regulation of gene expression at multiple levels by playing an active role in alternative splicing of pre-mRNAs (Bhardwaj et al., 2013; Lukavsky et al., 2013; Kuo et al., 2014), splicing of non-coding RNAs (Tollervey et al., 2011) and mRNA stability (Fukushima et al., 2019), thus affecting diverse cell processes, including mitochondrial homeostasis (Izumikawa et al., 2017), DNA damage response (Konopka et al., 2020; Riancho et al., 2020; Wood et al., 2020) and axonal transport (Fallini et al., 2012; Tripathi et al., 2014). While TDP-43 primarily localizes in the nucleus, upon cellular stress, it translocates to the cytoplasm to form stress granules together with other RBPs and stalled ribosomes (Colombrita et al., 2009). Interestingly, single cell proteomic analysis of somatic motor neurons (MNs) derived from *postmortem* spinal cords of ALS donors with TDP-43 pathology, not only facilitated the differentiation of disease states in individual MNs but also uncovered significant reduction in the abundance of proteins with critical roles in cell energetics, protein translation, proteostasis, and trafficking mechanisms such as Golgi-lysosome trafficking (Guise et al., 2024). Furthermore, *C9orf72* mutations lead to the formation of RNA foci which sequester a range of RBPs including TDP-43, resulting in its aberrant accumulation in the cytoplasm (Lee et al., 2013; Chew et al., 2019).

TDP-43 consists of four domains that mediate distinct activities: (a) a globular N-terminal domain (NTD) with a nuclear localization signal (NLS). The NTD is crucial for the formation of the TDP-43 functional dimer/oligomer (Qin et al., 2014; Mompean et al., 2016; Afroz et al., 2017; Jiang et al., 2017; Mompean et al., 2017) and recruits RNA for splicing (Jiang et al., 2017); (b) two tandem RNA recognition motifs (RRM1 and RRM2) (Lukavsky et al., 2013) with a nuclear export signal (NES) in RRM2 (Winton et al., 2008), although the NES functionality has been questioned (Archbold et al., 2018; Pinarbasi et al., 2018), and (c) a prion-like domain (PrLD) at the C-terminus encompassing two subdomains, a glutamine/asparagine-rich and a glycine-rich region, which is disordered and essential for protein-protein interactions (Buratti, 2020; Jiang et al., 2013). Six cysteine residues are present in TDP-43, with four located in the two RRM domains (Cys173, Cys175, Cys198, and Cys244), while the other two (Cys39 and Cys50) in the N-terminal domain (Valle and Carri, 2017). The oxidation of cysteines within the two RRMs decreases protein solubility and induces the formation of intra- and inter-molecular disulfide linkages (Cohen et al., 2012; Chang et al., 2013). The PrLD mediates TDP-43 intrinsic aggregation propensity (Johnson et al., 2009) and its incorporation into stress granules via its ability to undergo liquid-liquid phase separation (LLPS) (Conicella et al., 2016; Wang et al., 2018). Interestingly, the majority (∼95%) of the ALS-linked *TARDBP* mutations are localized at the C-terminal domain (Prasad et al., 2019); these TDP-43 variants show increased stress granule formation upon oxidative stress (Liu-Yesucevitz et al., 2010) and higher aggregation propensity (Conicella et al., 2016). Moreover, certain *TARDBP*-linked mutations enhance cytoplasmic mislocalization of TDP-43 (Barmada et al., 2010; Mutihac et al., 2015; Mitsuzawa et al., 2018). Finally, in addition to cysteine oxidation, also other PTMs such as phosphorylation, acetylation, ubiquitination and the proteolytic processing of TDP-43 that leads to the formation of C-terminal fragments are closely associated with the misfolding and aggregation of the protein (Buratti, 2018; Farina et al., 2021).

Cell culture and animal models have provided compelling evidence indicating a significant involvement of TDP-43 in the initiation and progression of motor neuron degeneration (Bonifacino et al., 2021). Transgenic mice overexpressing TDP-43 with familial fALS mutations, develop aggregates and manifest a full spectrum of ALS-like phenotypes at the molecular, cellular and behavioral levels (Ke et al., 2015; Huang et al., 2020). Similarly, the overexpression of a TDP-43 variant lacking the nuclear localization signal (dNLS), leads to the accumulation of insoluble, phosphorylated cytoplasmic TDP-43 in the brain and spinal cord. This is accompanied by brain atrophy, muscle denervation, significant motor neuron loss, and the development of progressive motor impairment (Walker et al., 2015). Notably, suppression of TDP-43 overexpression leads to a rapid clearance of TDP-43 pathology and rescues motor deficits in inducible mouse models (Ke et al., 2015; Walker et al., 2015). These collective findings strongly suggest that TDP-43 is a key protein implicated in neurodegenerative processes in motor neurons.

### The aggregation process

The term protein aggregation describes the transition of a protein from its native biologically functional state to the formation of oligomers and medium- or higher-order aggregates through self-association. Protein aggregation is usually caused by the presence of unfolded or misfolded species of the protein. Misfolded conformations typically expose hydrophobic segments within a hydrophilic- either intracellular or extracellular- environment, which subsequently tend to self-associate into soluble oligomers and eventually to larger insoluble aggregates. Most often, the building block of oligomeric and aggregated species is the protein’s unfolded/misfolded/partially folded monomer. The structure of such aggregates highly depends on the conformation of the monomeric state: disordered amorphous aggregates are usually formed by unfolded or native monomers while well-defined fibrils with cross-β structure (amyloid fibrils) can originate from partially folded monomers (Almeida and Brito, 2020). Amyloid deposits have been extensively implicated in disease pathogenesis, while there are few disorders associated with amorphous aggregates, such as cataracts caused by γD-crystallin disordered deposition (Moreau and King, 2012).

**FIGURE 1:**
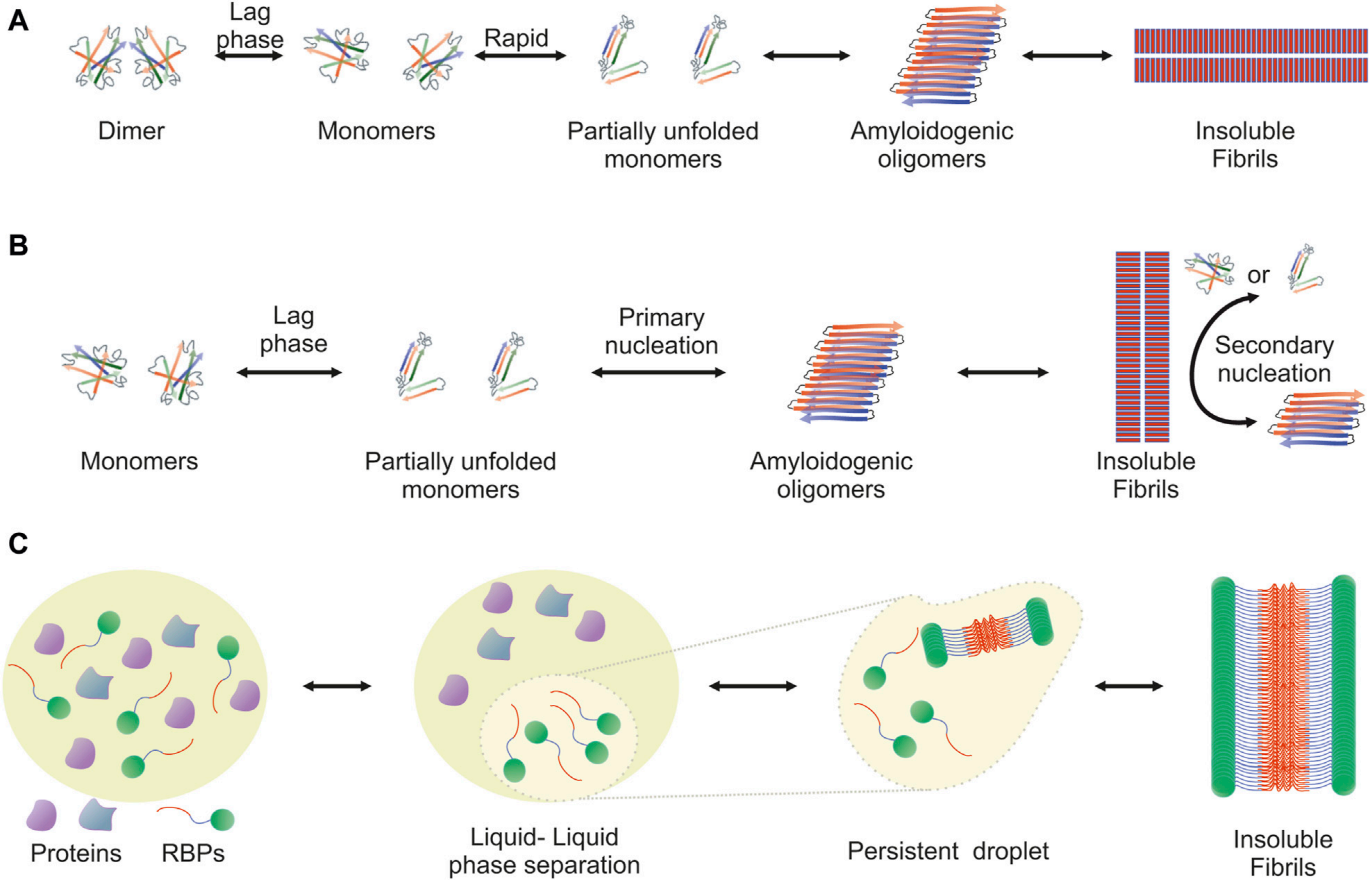
Visualization of the amyloidogenic pathway featuring three distinct models. Detailed overview of the diverse mechanisms through which amyloid fibrils can form, each highlighting different aspects and stages of the amyloidogenic process. **(A)** Downhill Polymerization Model: The protein first dissociates into native monomers, which is the rate-limiting step in the formation of fibrils. Subsequently, the monomeric species partially unfold to form the aggregation intermediates. Once such intermediates are formed, the following self-assembly process is a downhill polymerization. **(B)** Nucleation-Growth Model: Amyloid aggregation is kinetically dependent on nucleation events, which include primary and secondary nucleation. Primary nucleation refers to initial events where protein monomers associate into nucleation “seeds.” This is a rate-limiting, kinetically unfavorable process, but once the nuclei are formed, addition of further monomers is more favorable through an elongation process. Primary nucleation causes elongation into fibrils, which eventually leads to the formation of a critical mass of fibrils capable of catalyzing a secondary nucleation phase in which nucleus formation from monomers is catalyzed by existing aggregates. This creates a positive feedback loop of aggregation, where small and large oligomers are constantly formed. **(C)** Liquid-Liquid Phase Separation (LLPS) Model: It proposes that amyloidogenic proteins first undergo liquid-liquid phase separation, forming protein-rich droplets. Within these droplets, the concentration of proteins reaches a threshold that facilitates the transition from a disordered liquid state to a more ordered solid state, leading to amyloid fibril formation. This model provides insight into the spatial and temporal regulation of amyloidogenesis within cellular environments.

The amyloidogenic pathway has been extensively studied and two distinct mechanisms, the downhill polymerization and the nucleation-growth model, have been described. Both are characterized by sigmoidal kinetics and differ in the factor that determines the rate-limiting step. In each case, the mechanism of aggregation employed depends on the protein. Downhill polymerization (Figure 1A) is typically observed in proteins with oligomeric native conformation, such as transthyretin (TTR) (Hammarstrom et al., 2003; Eisele et al., 2015). The rate-limiting step is the disassembly of the native oligomer into unstable monomers, which then rapidly form aggregates (Lai et al., 1996; Hurshman et al., 2004; Eisele et al., 2015). Importantly, the lag phase is governed by the slow dissociation of native oligomeric state and seeding does not accelerate the aggregation process. The nucleation-growth model (Figure 1B), characteristic of the aggregation of amyloid-β peptide (Knowles et al., 2014), resembles the crystallization process (Teplow, 1998; Scherzinger et al., 1999; Wood et al., 1999; Knowles et al., 2014). The rate-limiting step for amyloid fibril development is the formation of protein oligomers (nuclei or seeds), which subsequently drive the rapid elongation of the fibrils by further monomeric or oligomeric species binding to the nuclei. The slow lag phase can be eliminated by adding pre-formed nuclei.

Another mechanism that leads to the formation of ordered aggregates is mediated by LLPS, typically observed in RBPs (Figure 1C). Approximately 30% of the genes associated with ALS encode RBPs, such as TDP-43, FUS, TATA-box binding protein associated factor 15 (TAF15) and proteins from the heterogeneous nuclear ribonucleoprotein (hnRNP) family, including hnRNPA1 and hnRNPA2B1 (Wroe et al., 2008). These proteins form cytoplasmic inclusions in motor neurons of ALS patients and their aggregation propensity is linked to the PrLDs they harbor (Molliex et al., 2015; Patel et al., 2015). PrLDs are a subset of intrinsic disordered regions (IDRs), characterized by low sequence complexity, comprising only a subset of the 20 amino acids; in particular, PrLDs are enriched in uncharged polar amino acids and glycine (Alberti et al., 2009). Intriguingly, about 30% of the 240 human proteins containing predicted PrLDs are involved in RNA processing (March et al., 2016; Verdile et al., 2019). LLPS describes liquid-liquid de-mixing: a high local concentration of IDRs promotes LLPS through transient and weak interactions among the IDRs of RBPs. Importantly, association of RBPs into “droplets” which are separated from the nucleoplasm or the cytoplasm is dynamic and transient. However, persistent association of RBPs with the separated liquid state results in the formation of stable hydrogels and eventually fibrils (Kato et al., 2012; Lin Y. et al., 2015; Guo and Shorter, 2015; Molliex et al., 2015; Murakami et al., 2015; Patel et al., 2015; Kato and McKnight, 2017; Shin and Brangwynne, 2017).

Until recently, there was intense debate over whether the protein aggregates detected in ALS patients are typical amyloids, primarily due to controversial findings. Initially, reports indicated that the TDP-43 positive filamentous aggregates found in ALS-affected motor neurons (Hasegawa et al., 2008; Lin and Dickson, 2008) did not stain with the amyloid-specific dyes Congo red and Thioflavin S (ThS) (Kerman et al., 2010). This finding was further supported by *in vitro* experiments, where recombinant TDP-43 formed Thioflavin T (ThT)-negative granulo-filamentous aggregates similar to those observed in ALS patients (Johnson et al., 2009) or amorphous aggregates (Capitini et al., 2014). On the other hand, it has been shown that peptide fragments from the C-terminal domain and certain TDP-43 variants form ThT-positive aggregates *in vitro*, acquiring a β-sheet-enriched structure (Chen et al., 2010; Guo et al., 2011; Zhu et al., 2014; Jiang et al., 2016). These conflicting findings can now be explained based on the structure of TDP-43 aggregates from ALS with FTLD (Arseni et al., 2022), revealing the formation of filaments which are structurally distinct from cross-β amyloid fibrils and a novel fold that bears no similarity to TDP-43 filaments formed *in vitro* (Cao et al., 2019; Li et al., 2021).

The Falcon group employed cryogenic electron microscopy to determine the structures of aggregated TDP-43 in the frontal and motor cortices of an individual who succumbed to ALS with FTLD, as well as in the frontal cortex of another individual with the same diagnosis (Arseni et al., 2022). Remarkably, they identified an identical amyloid-like filament structure consisting of a single protofilament in both brain regions and individuals. The ordered filament core, spanning residues 282 to 360 in the TDP-43 low-complexity domain, adopts a unique double-spiral-shaped fold. The filament structure lacks the typical β-sheet stacking associated with cross-β amyloid formation due to an abundance of glycine and neutral polar residues, facilitating numerous turns and restricting β-strand length. The uneven distribution of residues results in structurally and chemically distinct surfaces, suggesting potential ligand binding sites on external densities.

This study also revealed that the brain-derived TDP-43 filaments are structurally distinct compared to filaments assembled *in vitro* from its LCD under mildly acidic conditions (pH 4), or related fragments (Li et al., 2021). These differences include opposite chirality and variations in both protein fold and secondary structure. For the LCD, the structure revealed single protofilament fibrils featuring a large core comprised of 139 residues out of the 148 present in the LCD, tightly packed together. The C-terminal segment of this core exhibits mainly planar characteristics and is marked by a small proportion of hydrophobic amino acids. In contrast, the N-terminal region includes numerous hydrophobic residues and adopts a non-planar backbone conformation, leading to the rugged surfaces observed at the ends of the fibrils.

Cryo-electron microscopy was also employed to elucidate the structures of two segments identified as the pathogenic cores in human TDP-43 aggregation (Cao et al., 2019). The first segment, SegA (residues 311–360), exhibits three polymorphs, each characterized by a dagger-shaped fold, spanning residues 312–346. Tight hydrophobic interactions are formed by hydrophobic residues ranging from Phe313 to Ala341 whereas the dagger tip is created by a sharp 160° kink at Gln327. The variations among the three SegA polymorphs primarily arise from differences in protofilament numbers and symmetry. In contrast to the polymorphic nature of SegA, the second segment, SegB A315E (residues 286–331, containing the ALS hereditary mutation A315E), forms fibrils with a consistent morphology. These fibrils adopt an R-shaped fold spanning residues 288–319, which, overall, displays a more pronounced kink compared to the dagger-shaped fold, likely attributable to the higher prevalence of glycine residues. Each fibril is composed of four protofilaments, and notably, all four R-folds are characterized by the presence of a salt-bridge between Arg293 and Glu315, facilitated by the pathogenic A315E mutation. This salt-bridge may impact the kinetics of fibril growth and nucleation, providing a mechanistic explanation for A315E’s propensity to promote TDP-43 aggregation through electrostatic attraction with Arg293.

Along the same lines, SOD1-positive inclusions from ALS cases display a fibrillary morphology (Kato et al., 2000) but do not react with ThS (Kerman et al., 2010). In contrast, SOD1 cytoplasmic aggregates from a transgenic SOD1-fALS mouse model could be stained with ThS (Furukawa et al., 2008). Importantly, the aggregation kinetics of SOD1 are typically monitored by ThT staining (Rakhit et al., 2002; Furukawa et al., 2008; Iwakawa et al., 2017; Shvil et al., 2018) and pre-formed SOD1 aggregates exhibit seeding activity both *in vitro* and intracellularly (Furukawa et al., 2008; Furukawa et al., 2013). Moreover, ALS-related SOD1 mutant proteins crystallize in forms comprising higher-order assemblies of aligned β-sheets (Elam et al., 2003). On the other hand, *in vitro* monitoring of SOD1 aggregation has demonstrated that a competition between amorphous and amyloid aggregation occurs (Abdolvahabi et al., 2016), proposing a possible factor for the wide variability in kinetic results among publications (Crown et al., 2019).

In a recent study, cytotoxic amyloid fibrils were generated from full-length human apo-SOD1 under reducing conditions, and their atomic structure was elucidated using cryo-EM (Wang LQ. et al., 2022). The SOD1 fibril is composed of a singular protofilament featuring a left-handed helix. Within the fibril core, a serpentine fold is formed, incorporating six β-strands of the N-terminal segment (residues 3–55) and seven β-strands of the C-terminal segment (residues 86–153), with an unstructured region in between. This novel amyloid fibril structure displays a very compact fold, and the connection of the two segments is stabilized by three pairs of salt bridges, effectively “zipping up” the structure.

### Characteristics of oligomeric species

A multitude of pathogenic mechanisms have been described for ALS, including protein misfolding and aggregation, glutamate excitotoxicity, oxidative stress, mitochondrial dysfunction, declined autophagy, neuroinflammation and DNA damage. However, the drive behind ALS onset and progression, as well as the degree to which each mechanism contributes to disease phenotypes and heterogeneity, remains unclear. Current evidence supports that protein misfolding and aggregation exert toxic effects on cellular fitness, eventually compromising organismal health. Regarding the toxic agent in ALS and other conformational neurodegenerative diseases, the debate continuous on whether soluble misfolded species (monomeric or oligomeric) or insoluble aggregates confer toxicity (Gelpi and Colom-Cadena, 2019).

Recent work has highlighted SOD1 oligomers, particularly the trimeric variants, as the neurotoxic entities (Proctor et al., 2016). The study revealed that SOD1 mutants promoting trimerization led to increased neuronal cell death and established a direct association between misfolded oligomers and neuron death by linking cytotoxicity to trimer stability. Additional studies showed that large aggregates are non-toxic and actually play a neuroprotective role (Zhu et al., 2018; Gill et al., 2019). Interestingly, SOD1 mutants designed to promote the formation of large aggregates and destabilize toxic trimers did not impact cell viability (Zhu et al., 2018). The mechanism leading to the formation of non-native trimers remains unclear, however, in order to occur, SOD1 must undergo dissociation into monomers (Khare et al., 2004). External factors, such as oxidative stress (Wilcox et al., 2009) or exposure to the toxin β-methylamino-L-alanine (BMMA) (Proctor et al., 2019), may play a role in the transition of SOD1 dimers into monomers. At present, there are two hypotheses regarding trimer formation (Choi and Dokholyan, 2021). The first suggests that trimers occur on the pathway from monomers to native dimers (dissociating into monomers), then to trimers and finally to larger aggregates. The second posits that trimer formation is an off-pathway phenomenon, meaning it does not lead to the generation of large aggregates, and current experimental evidence favors this scenario (Hnath and Dokholyan, 2022). Indicatively, the abundance of soluble misfolded SOD1 subfractions, in the range from monomeric to trimeric in size, has been associated with a reduced lifespan in SOD1-ALS mouse models (Zetterstrom et al., 2007).

Despite the association of TDP-43 aggregation with disease (Neumann et al., 2006; Hasegawa et al., 2008), several studies have highlighted the functional oligomerization of TDP-43 in physiological contexts. The NTD residues 1–105 and 1–265 induce the formation of homo-oligomeric species in a concentration-dependent manner, initiated by the formation of intermolecular disulfide bonds ultimately leading to its tetramerization (Chang et al., 2012; Jiang et al., 2017). Oligomerization of TDP-43 is crucial for its nuclear RNA splicing activity and, interestingly, serves to prevent the aggregation of the C-terminal TDP-43 within the nucleus of neuronal cells (Afroz et al., 2017; Jiang et al., 2017). The crystal structure of a purified recombinant human TDP-43 fragment (residues 1–80) was determined at a resolution of 2.1 Å, revealing a unique pattern of head-to-tail interactions between monomers, resulting in the formation of solenoid-like polymers (Afroz et al., 2017). In contrast to physiological oligomers, pathological TDP-43 oligomers exhibit distinct characteristics. Detectable in the early stages of aggregation, their formation is accelerated in the presence of disease-associated TDP-43 mutations (Johnson et al., 2009; French et al., 2019). Using a polyclonal TDP-43 oligomer-specific antibody (Fang et al., 2014), pathological TDP-43 oligomers were identified in FTLD patients (Kao et al., 2015).

The TDP-43 oligomers manifest as a diverse spectrum of molecules, ranging from low-molecular-weight species like dimers, trimers, and tetramers, to a variety of high-molecular-weight species (Johnson et al., 2009; Guo et al., 2011; Choksi et al., 2014; Fang et al., 2014; French et al., 2019). Described as heterogeneous structures at the intermediate stage of aggregate formation, the oligomerization process may involve cysteine oxidation, particularly in the initial stages (Bozzo et al., 2016). Extracellular exposure to TDP-43 oligomers demonstrated cytotoxicity in neuroblastome cells (Choksi et al., 2014), and intrahippocampal injection of these oligomers caused damage to hippocampal neurons in wild-type mice (Fang et al., 2014), further substantiating their neurotoxicity. Interestingly, in an animal model expressing the ALS-related TDP-43 (M377V) variant at nearly endogenous levels, there was complete absence of mature insoluble aggregates (Gordon et al., 2019). However, these mice progressively developed motor function deficits concomitant with the loss of neuromuscular junction integrity, thus recapitulating ALS phenotypes (Gordon et al., 2019). Taken together, current evidence is not sufficient to argue towards one or the other direction, though it seems possible that both oligomeric soluble species and insoluble aggregates contribute differentially to ALS pathogenesis (Arnold et al., 2013; Gordon et al., 2019).

The ability to isolate, cultivate, and reprogram cells obtained from patients, such as fibroblasts, into central nervous system (CNS) cells, has introduced a novel approach that complements traditional preclinical *in vitro* and *in vivo* models (Ferraiuolo and Maragakis, 2021; Du et al., 2023). There are two primary approaches to this method. Several laboratories have investigated the use of induced pluripotent stem cells (iPSCs), focusing on generating motor neurons from patients with mutations in *TARDBP*, *FUS*, *SOD1*, and *C9orf72*, or those with sporadic disease (Ferraiuolo and Maragakis, 2021). For instance, motor neurons derived from ALS patients with mutated TDP-43, developed cytosolic aggregates resembling those observed in *postmortem* tissues and displayed shorter neurites (Egawa et al., 2012). Importantly, the accumulation of insoluble protein inclusions has been observed in numerous studies using iPSC-derived motor neurons (Dimos et al., 2008; Burkhardt et al., 2013; Chen et al., 2014; Seminary et al., 2018), although neuronal cell death is not observed, supporting the non-cell-autonomous hypothesis of ALS neurodegeneration (Ilieva et al., 2009; Wachter et al., 2015).

Limiting the screening approach to monocultured motor neurons restricts the influence of non-neuronal cells on observed phenotypes. In ALS, neighboring cells like astrocytes and microglia significantly impact neurodegenerative phenotypes and neuronal survival (Boillee et al., 2006). Additionally, iPSC technology often results in the loss of the cellular aging signature (Lapasset et al., 2011; Patterson et al., 2012). An alternative method involves trans-differentiating patient fibroblasts into neural progenitors, which can then be further differentiated into neurons or astrocytes (Meyer et al., 2014; Mertens et al., 2016; Tang et al., 2017). These astrocytes exhibit expected toxicity toward co-cultured motor neurons and seem to maintain the associated aging phenotype (Gatto et al., 2021). Interestingly, a study using iPSC-derived motor neurons and astrocytes revealed that recombinant TDP-43 oligomers, can induce neuronal toxicity, leading to increased caspase-3 activity and to apoptosis of neurons but not astrocytes (Smethurst et al., 2020).

### Mechanisms of aggregation-mediated toxicity

Concerning the mechanisms underlying aggregation-mediated toxicity in ALS and other protein misfolding diseases, two major models have been proposed: the loss-of-function hypothesis and the gain-of-toxic activity hypothesis. While both terms are borrowed from genetics, mutations are not always relevant within the context of misfolding and aggregation. In the majority of ALS cases, misfolded species originate from a wild-type protein devoid of inherited mutations. Thus, in this section we focus on how the aggregation process itself, rather than the genetic background (as reviewed in Kim et al., 2020), can lead to phenotypes resembling loss and gain of activity. Importantly, these mechanisms are not mutually exclusive; in fact, some alterations observed in ALS-related cellular physiology can be better explained by a combination of loss and gain of activity.

#### The loss-of-function hypothesis

In genetics, the term “loss-of-function” is used to describe a mutation that partially or completely disrupts the activity of a gene product. Accordingly, during the aggregation process, sequestration and often mislocalization of a protein within aggregates, reduce its abundance, thereby leading to impaired activity. Indicatively, most ALS-affected motor neurons exhibit wild-type TDP-43 cytoplasmic inclusions concomitant with TDP-43 clearance from the nucleus, indicating that a loss of TDP-43 splicing activity could contribute to disease phenotypes (Polymenidou et al., 2011; Tollervey et al., 2011). In support of this, *Tardbp* knockout mice display early embryonic lethality (Kraemer et al., 2010); moreover, TDP-43 is essential for normal motor neuron function in flies (Feiguin et al., 2009) and mice (Yang et al., 2014), as evidenced by motor dysfunction and deficits in the neuromuscular junction after TDP-43 depletion. Conditional *Tardbp* knockout mice, when crossed with various tissue-specific Cre lines, exhibit diverse cellular and behavioral phenotypes, ranging from electrophysiological abnormalities to deficits in motor movement (Iguchi et al., 2013; Wu et al., 2019). TDP-43 has been demonstrated to interact with RNA transcripts of over 6,000 genes in mice. Accordingly, the reduction of TDP-43 levels in adult mouse brains using antisense oligonucleotides (ASOs) led to the differential regulation of approximately 600 genes, mostly involved in synaptic activity and neuronal development, and to the alteration of 1,000 splicing events (Polymenidou et al., 2011).

Loss of function, at least partially, can also be attributed to SOD1-linked ALS. Although *Sod1* knockout mice (*Sod1* ^*−/−*^) develop normally and do not display evident ALS-like phenotypes (Reaume et al., 1996), they do exhibit- together with other pathologies-progressive motor neuropathy characterized by peripheral motor axon degeneration (Flood et al., 1999; Fischer et al., 2011; Larkin et al., 2011; Fischer et al., 2012; Shi et al., 2014; Ivannikov and Van Remmen, 2015). Furthermore, recent human genetics studies indicate that SOD1 homozygous loss-of-function can lead to debilitating phenotypes, including progressive loss of motor abilities, tetraspasticity, and hyperreflexia, but not ALS. In contrast, heterozygous carriers remained unaffected (Andersen et al., 2019; Park et al., 2019).

#### The gain-of-toxic activity hypothesis

The accumulation of insoluble proteins is a prominent feature in ALS pathology, suggesting an interconnection between protein aggregation and disease development. The hypothesis posits that protein aggregates may initiate the disease process by acquiring toxic properties, which are mainly exerted through the sequestration of crucial components of physiological processes, such as RNA metabolism, DNA damage response, synaptic signaling and axonal trafficking, thereby disrupting these cellular functions and contributing to disease progression (Ilieva et al., 2009). For instance, TDP-43 aggregates have been found to contain proteins like the RNA-processing factor Matrin 3 (MATR3) (Tada et al., 2018), the ubiquitin-proteasome system factor Ubiquilin 2 (UBQLN2) (Deng et al., 2011; Williams et al., 2012), the Golgi complex and membrane trafficking factor Optineurin (OPN) (Hortobagyi et al., 2011) and the Rho guanine nucleotide exchange factor 28 (RGNEF) (Keller et al., 2012) involved in synapse formation and dendritic morphogenesis. Other potential mechanisms for the induction of cell toxicity by protein aggregation in ALS include disruption of the proteostasis network, leading to impairment of crucial protein degradation pathways like the ubiquitin proteasome system and autophagy (Medinas et al., 2017; Ramesh and Pandey, 2017; Montibeller et al., 2020). Compelling evidence also connects aggregated SOD1 and TDP‐43 to mitochondrial dysfunction and degeneration (Jankovic et al., 2021). TDP-43 aggregates sequester specific microRNAs and mitochondrial proteins encoded by the nuclear genome, with dysregulation of their expression levels leading to mitochondrial dysfunction and oxidative stress (Zuo et al., 2021). Aggregates of mutant SOD1 in the intermembrane space of mitochondria diminish the activity of the electron transport chain (ETC) complexes in rats (Pickles et al., 2013) and affect mitophagy by disabling recruitment of autophagy receptors on damaged mitochondria in N2a cells (Tak et al., 2020).

Alternatively, aberrant protein aggregates can induce a chronic inflammatory response in the brain contributing to disease progression (Appel et al., 2021). In a recent study, Yu et al. demonstrated that TDP-43 induces inflammation in ALS by initiating the release of mitochondrial DNA into the cytoplasm. This, in turn, activates the cytoplasmic DNA-sensing cyclic GMP-AMP synthase (cGAS)/stimulator of interferon genes (STING) pathway (Yu et al., 2020). Separately, the nuclear factor-kappa β (NF-κB) protein has been reported as a master regulator of inflammation in ALS (Haidet-Phillips et al., 2011); in mutant SOD1 mice, NF-κB signaling becomes activated within glia as the disease progresses (Frakes et al., 2014). In support of these findings, extensive astrocytosis (Kushner et al., 1991; Nagy et al., 1994; Reischauer et al., 2018; Schiffer et al., 1996), microglial activation (Henkel et al., 2004; Turner et al., 2004; Zurcher et al., 2015), as well as increased levels of inflammatory cytokines (Moreau et al., 2005; Liu et al., 2015; Lu et al., 2016) and elevated levels of chitotriosidase (Chit-1) and chitinase-3-like protein 1 (CHI3L1) in cerebrospinal fluid (CSF) correlated to disease progression (Vu et al., 2020), have been detected in ALS patients.

## Therapeutic targeting of misfolding and aggregation

Among neurodegenerative diseases, ALS stands out as one of the few for which disease-modifying therapies have gained approval. The current standard of care for ALS patients involves multidisciplinary symptom management, both pharmacological and non-pharmacological, such as nutritional and respiratory support (Hardiman et al., 2017; Mejzini et al., 2019). Clinical trials in ALS have assessed over 60 compounds (Petrov et al., 2017), each with distinct mechanisms of action; however, only four- riluzole, edaravone, AMX0035 and tofersen- have been granted regulatory clearance for clinical use. Riluzole, the first FDA-approved ALS therapy in 1995 (Lacomblez et al., 1996a; Lacomblez et al., 1996b), is not a cure for ALS. Instead, it is a neuroprotective drug that decreases glutamate release into the synaptic cleft by blocking voltage-gated sodium channels on presynaptic neurons, thereby mitigating excitotoxicity (Wang et al., 2004; Bissaro and Moro, 2019). While Riluzole does not modify the course of the disease (Fang et al., 2018), it prolongs the patient’s survival from 6 to 19 months (Andrews et al., 2020). Edaravone is a potent anti-oxidant and a free-radical scavenger, preventing oxidative stress-induced motor neuron death (Jami et al., 2015; Soejima-Kusunoki et al., 2022). A randomized controlled trial conducted in Japan demonstrated the effectiveness of Edaravone in slowing the rate of motor function deterioration, particularly in selected patients with early disease onset and rapid progression (Writing Group and Edaravone MCI-186 ALS 19 Study Group, 2017). Its impact on survival is yet to be determined. Apart from Japan and other Asian countries, its use has been approved by the FDA and Health Canada, but not yet from the EMA. A recently developed oral formulation in the United States is expected to replace the intravenous version (Pattee et al., 2023). AMX0035 was approved in 2022 for the treatment of ALS in the United States and Canada, and it is currently being evaluated in the PHOENIX phase III clinical trial (Nikitin et al., 2023). It is a coformulation of sodium phenylbutyrate, a chemical chaperone that improves endoplasmic reticulum (ER) folding capacity by upregulating heat-shock proteins (Suaud et al., 2011), and taurursodiol (tauroursodeoxycholic acid), an inhibitor of mitochondrial-associated apoptosis (Rodrigues et al., 2003). This therapeutic approach is designed to address various ALS-associated pathophysiological mechanisms such as mitochondrial dysfunction and ER stress that ultimately lead to motor neuron injury and cell death.

Tofersen is an antisense oligonucleotide (ASO) that obtained its initial approval in the United States on April 25, 2023, and in Europe on February 22, 2024, for the treatment of ALS in patients with a confirmed *SOD1* mutation. It has been designed to mediate RNase H–dependent degradation of *SOD1* mRNA to reduce the synthesis of SOD1 protein (McCampbell et al., 2018; Rinaldi and Wood, 2018). The preclinical rationale supporting the ASO knockdown of SOD1 was very robust, stemming from several years of systematic analysis showing increased survival and enhanced motor performance in SOD1 (G93A) rats and mice and decreased SOD1 protein levels in nonhuman primates (Iannitti et al., 2018; McCampbell et al., 2018). Its intrathecal administration in clinical trials demonstrated a deceleration in the decline of participants with rapidly progressing disease and apparent clinical stabilization in those with slower progressing disease (Miller et al., 2022). During the intervention period, concentrations of phosphorylated neurofilament heavy and neurofilament light (NFL) in plasma and CSF, biomarkers indicative of axonal injury and neurodegeneration, were found to be decreased (Miller et al., 2022). Tofersen is currently being evaluated in the phase III ATLAS study for its ability to delay the clinical onset of ALS in pre-symptomatic individuals with a confirmed SOD1 mutation (Benatar et al., 2022).

The complex nature of ALS genetics, coupled with the disease’s high heterogeneity, presents a great challenge in developing treatment strategies that universally benefit every patient. Consequently, there has been a shift in focus towards identifying converging paths and common pathologies that contribute to motor neuron vulnerability and degeneration in ALS. Among these factors, protein aggregation emerges as a prevalent underlying cause not only in ALS but also in various other neurodegenerative diseases. Despite the diversity of mutations in different genes, leading to potential variations in the aggregated proteins, the shared problem of protein aggregation spans a wide spectrum of patients, encompassing familial ALS (fALS), sporadic ALS (sALS), and ALS with frontotemporal dementia (ALS/FTLD) (Blokhuis et al., 2013). As a result, the pursuit of therapies targeting protein aggregation holds significant promise and represents a crucial step forward in addressing ALS and related disorders (Elliott et al., 2020).

Several approaches have been applied to reduce the levels of toxic variants; these include proteolysis targeting chimeras (PROTACs) inducing protein degradation (Tseng et al., 2023), antibodies (Maier et al., 2018; Pozzi et al., 2019; Afroz et al., 2023; Audrain et al., 2023; Bakavayev et al., 2023), vaccines (Zhao et al., 2019), antisense oligonucleotides (Iannitti et al., 2018; Mead et al., 2023), RNA interference (RNAi) with small RNAs (shRNA and miRNA) (Bravo-Hernandez et al., 2020; Mueller et al., 2020), and CRISPR/Cas9 gene editing (Duan et al., 2020). Another way of reducing the accumulation of toxic aggregates is to restore dysfunctional proteostasis by upregulating chaperones (Kalmar and Greensmith, 2017; Kinger et al., 2023), inducing autophagy (Chen et al., 2020) and activating the proteasome (Webster et al., 2017). Currently, many compounds targeting proteostasis, such as trehalose (Seelos Therapeutics) and Trametinib (GENUV) are being explored in clinical trials. Moreover, PMN-267 (ProMIS Neurosciences), an antibody that targets the formation of misfolded TDP-43, is under preclinical development (Mead et al., 2023). Despite the success of Tofersen, a similar strategy utilizing intrathecally delivered ASOs for C9orf72-associated ALS did not show clinical benefits, leading to the termination of the clinical trial in 2022 (Mead et al., 2023).

In this review, we focus on molecules that directly target the misfolded species (monomers, oligomers or mature aggregates) to (a) **stabilize** the native protein conformation, (b) **restore** the native conformation of misfolded species and (c) inhibit protein **oligomerization**. Such molecules fall into two categories depending on the specificity to bind to their target, namely, chemical and pharmacological chaperones.

The term “chaperone” is borrowed from the name of a class of cellular proteins that assist polypeptide chains in acquiring their native, functionally active, conformation. They participate in (a) the folding of nascent polypeptides (*de novo* folding) or the refolding of stress-mediated misfolding, (b) the disaggregation of protein aggregates and (c) proteostatic pathways that direct denatured and misfolded/unfolded proteins for degradation (Wentink and Rosenzweig, 2023). However, pharmacological chaperones, unlike proteins, are low molecular weight chemical molecules that exert their action by specifically binding to their target proteins. They typically stabilize an already folded or partially folded protein, protect it from thermal denaturation, prevent proteolytic degradation and also inhibit aggregation, restoring proper steady-state levels (Convertino et al., 2016; Bose and Cho, 2017). Pharmacological chaperones have successfully been used experimentally *in vitro* and *in vivo* (Leidenheimer and Ryder, 2014; Hou et al., 2018), and they have entered clinical trials, to restore the function of specific misfolded proteins (Liguori et al., 2020; Tran et al., 2020). For example, Tafamidis has been approved for the treatment of transthyretin amyloidosis (ATTR) (Bulawa et al., 2012; Maurer et al., 2018) and Migalastat has been licensed for the treatment of Fabry disease (FD) in patients carrying specific lysosomal enzyme α-galactosidase A (GLA) variants (Germain et al., 2016).

Chemical chaperones are also low-molecular-weight compounds that lack specificity since they can bind to and stabilize virtually any protein without having a designated binding site (Cortez and Sim, 2014). They can be divided into two groups: osmolytes and hydrophobic compounds. Osmolytes, such as glycerol, produce a hydrophobic environment around proteins by removing water molecules, thereby increasing the free energy of the unfolded state and eventually shifting the equilibrium towards the folded state (Wang and Bolen, 1997; Street et al., 2006). Hydrophobic chaperones facilitate proper folding by directly interacting with the exposed hydrophobic regions of unfolded proteins, resembling the action of molecular chaperones (Kuzuhara et al., 2008). For example, the green tea catechin, (−)-epigallocatechin gallate (EGCG), seems to exert its general anti-amyloidogenic properties by directly binding to unfolded/misfolded proteins (Debnath et al., 2016). As mentioned above, the best-known example of chemical chaperone for ALS treatment is sodium phenylbutyrate (Suaud et al., 2011).

### Discovering inhibitors of protein misfolding and aggregation

Developing a new drug is a complex and challenging process that yields significant benefits for both society and the scientific community. High-throughput screening (HTS) is crucial in the early stages of small-molecule drug development, particularly when insufficient information limits structure-based approaches (Blay et al., 2020). For targets such as oligomeric species and aggregates, which lack well-defined structures and exist in dynamically varying mixtures, HTS requires innovative strategies to discover effective treatments. The utilized methods must be biologically relevant, sensitive, robust, and cost-effective, targeting diseases of significant relevance. HTS integrates biochemical and cell-based assays with computational strategies to identify potential drug candidates. *In silico* screening employs computational methods to predict interactions between molecules and targets, a critical step given the extensive chemical space. Unlike traditional HTS, which assesses thousands of compounds, virtual screening has the capacity to evaluate billions (Bohacek et al., 1996; Gorgulla et al., 2022), showcasing the expansive scope of modern drug discovery efforts.

In 2005, Ray et al. employed an *in silico* screening strategy of approximately 1.5 million compounds from commercial libraries (Ray et al., 2005). The objective was to identify molecules capable of stabilizing the SOD1 (A4V) dimer. The screening process yielded 100 hits, and subsequent mutagenesis studies using various *in vitro* aggregation assay protocols helped narrow down the selection to the 15 most effective compounds. Notably, three of the top five compounds shared pyrimidine-like structures. However, subsequent co-crystal structures of SOD1 with various small molecules and pyrimidine-like compounds challenged the initial assumption that the observed protection was solely due to binding to the hydrophobic cavity formed at the dimer interface. Instead, these structures suggested that the ligands interacted with Trp32, offering new insights into the mechanism of action (Antonyuk et al., 2010; Wright et al., 2013).

Nowak et al. developed (Nowak et al., 2011) an algorithm for the *in silico* screening of an extensive library comprising 2.2 million small molecules sourced from 11 commercially available databases. The primary objective was to discern compounds exhibiting selective binding affinity for mutant SOD1 over plasma proteins. Their investigation revealed a substantial subset of compounds displaying robust binding capabilities to SOD1, particularly within a hydrophobic cavity encompassing Val7–Gly147–Val148 at the dimerization interface. Notably, *in vitro* experimentation demonstrated the pronounced inhibitory effects of both isoproterenol and 5‐fluorouridine on the aggregation process of mutant SOD1.

In another computational screen involving 4,400 drugs and compounds, three flavonoids, quercitrin, quercetin-3-β-D-glucoside (Q3BDG) and EGCG, were predicted to bind around the dimer interface and stabilize SOD1, potentially inhibiting its aggregation, predictions which were confirmed experimentally (Ip et al., 2017). Interestingly, quercitrin and Q3BDG outperformed EGCG both *in silico* and *in vitro*. The tripeptide CGH (Srinivasan and Rajasekaran, 2019), hesperidin and 2,3,5,4′-tetrahydroxystilbene-2-O-β-D-glucoside retrieved from the traditional Chinese medicine database (Huang et al., 2014), and the natural polyphenols, kaempferol, and kaempferide (Srinivasan and Rajasekaran, 2018) have all been predicted to bind SOD1 and inhibit its aggregation, necessitating experimental verification.

#### Biochemical screens

This type of screens involves the use of a purified target protein of interest to assess the aggregation-inhibitory activity of test compounds. To quantify the outcomes of these assays, various optical methods such as absorbance, fluorescence, or luminescence are employed as readouts (Fang, 2012). For example, a screening assay of 640 FDA-approved drugs, aiming to evaluate their impact on the abnormal oligomerization of SOD1 (G37R) has been conducted *in vitro*. The primary objective was to identify molecules capable of inhibiting the formation of insoluble disulfide-linked oligomers. Among the extensive drug repertoire, six compounds - simvastatin, lovastatin, mevastatin, miltefosine, alfacalcidol and calcitriol-exhibited remarkable efficacy in almost completely suppressing the increase in solution turbidity (Anzai et al., 2016).

#### Cellular screens

Cell-based assays play a crucial role at every stage of the drug discovery process, serving various purposes from target identification and validation to lead optimization and safety screening (Macarron and Hertzberg, 2009; McGown and Stopford, 2018). For ALS, cell models employed in these assays include the NSC-34 cell line (Barber et al., 2009), created by fusing MN from the spinal cords of mouse embryos with mouse neuroblastoma cells, human astrocyte-derived H4 cells (Murakami et al., 2011), and PC12 cells from the rat adrenal gland (Benmohamed et al., 2011; Xia et al., 2011; Zhang W. et al., 2012; Wright et al., 2012; Boyd et al., 2014). Emphasis has also been given to the use of primary rat MN cultures (Vincent et al., 2005) or mouse embryonic stem cell‐derived MNs in small molecule screening studies (Yang et al., 2013; Thams et al., 2019). Finally, the use of iPSC technology has significantly contributed to the development of current ALS drug-screening platforms (Burkhardt et al., 2013; Imamura et al., 2017).

**FIGURE 2:**
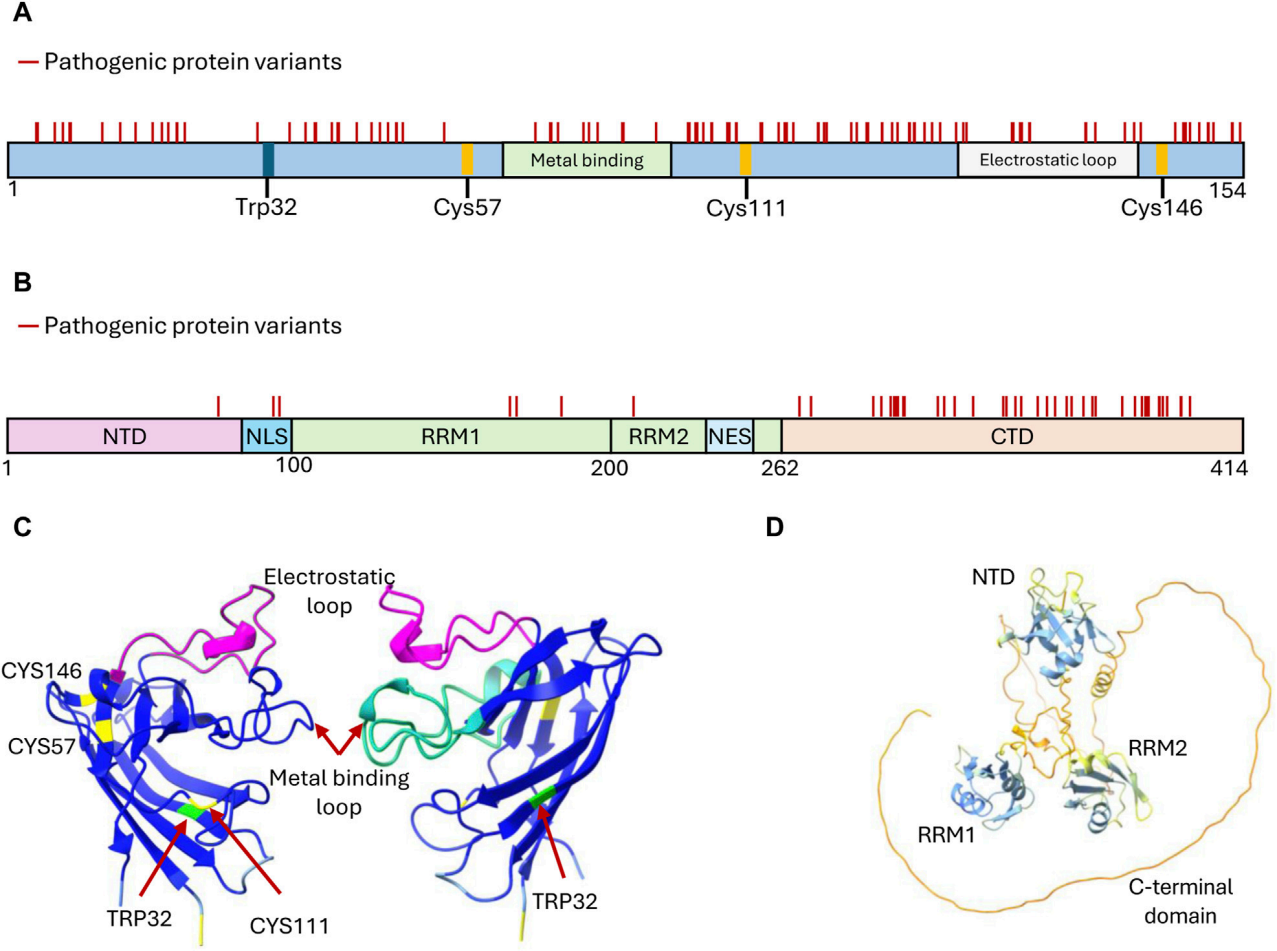
Structural features of SOD1 and TDP-43 proteins targeted by developed aggregation inhibitors. **(A)** Domain organization of SOD1 depicting structural features critical for its function, i.e., the cysteine residues participating in disulfide bond formation (Cys57 and Cys146), the metal-binding domain, and the electrostatic loop. These sites, along with Trp32 and Cys111, have been explored for drug discovery targeting SOD1 inhibition of aggregate formation. Mutations that generate amino acid substitutions in SOD1 are depicted with solid lines. **(B)** Domain organization of TDP-43 depicting the features that mediate functional oligomerization (N terminal domain-NTD), RNA binding (RNA Recognition motifs RRM1 and RRM2), protein interactions and liquid-liquid phase separation (C terminal domain-CTD), and nucleo-cytoplasmic shuffling (Nuclear Localization Signal-NLS and Nuclear Export Signal-NES). Mutations leading to amino acid substitutions in TDP-43 are represented by solid lines. **(C)** Structure of two SOD1 subunits rotated 180° to showcase the arrangement of key residues and domains (Uniprot P00441). Reconstituted using Chimera X (AlphaFold). **(D)** Structure of TDP-43 (Uniprot Q13148) highlighting the arrangement of its domains. Predicted AlphaFold structure reconstituted using Chimera X (Pettersen et al., 2021).

In the following section, we describe molecules that have emerged from drug-screens based on the above platforms and target misfolded species of SOD1 and TDP-43 (Figure 2).

## Molecules targeting SOD1 misfolding and aggregation

### Stabilization of the dimer

Upon translation, SOD1 undergoes spontaneous folding, primarily initiated around hydrophobic residues, culminating in the formation of a monomeric Greek-key β-barrel structure. For SOD1 to achieve activity and stability, three essential PTMs are required. The binding of zinc and the formation of an intra-subunit disulphide bond establish the SOD1 homodimer interface in conjunction with the N- and C-termini and four inter-subunit hydrogen bonds are formed. The introduction of copper to the SOD1 active site enhances thermal stability and activates enzymatic functionality (Hornberg et al., 2007). In the absence of these PTMs and the subsequent homodimer formation, SOD1 exhibits diminished thermal stability, populates unfolded conformations, and is susceptible to self-association (Culik et al., 2018). Interestingly, the engineering of an inter-subunit disulfide bond in the SOD1 (A4V) variant alleviated aggregation and restored enzymic activity (Ray et al., 2004), suggesting that stabilizing the dimer is a promising therapeutic strategy (McAlary and Yerbury, 2019; Yerbury and Cashman, 2020).

#### Targeting the Cys111 residue

The covalent attachment of small molecules to Cys111 has been a frequently employed strategy in the discovery of pharmacological chaperones for SOD1. This is attributed to their accessibility, reactivity, and potential for linking or stabilizing the SOD1 homodimer, as opposing Cys111 residues are separated by a short distance across the dimer interface and possess a highly reactive sulfhydryl group. Tethering Cys111 residues with maleimide linkers to bridge the 9 Å gap across the SOD1 dimer interface substantially increases the thermal stability of the SOD1 (G93A) and SOD1 (G85R) ALS-linked variants, restoring the enzymatic activity and metal-binding ability of the otherwise inactive G85R variant (Auclair et al., 2010). However, maleimide compounds do not exhibit favorable drug-like properties (Gavrin et al., 2012), while maleimide crosslinking inhibits SOD1 complex formation with the human copper chaperone for SOD1 (CCS), an interaction essential for SOD1 activation (Wright et al., 2016). Similarly, cyclic disulfides have demonstrated the ability to link opposing Cys111 residues and exhibited cell penetrability (Wright, 2020). For example, mono-S-oxo derivatives were able to bind and tether 95% of SOD1 monomers as dimers within cells (Donnelly et al., 2018). Taking a different approach, cisplatin enhances the thermal stability of metal-free SOD1 and can effectively prevent or reverse SOD1 aggregation within cells without the need for subunit tethering (Banci et al., 2012) but hinders complex formation between SOD1 and hCCS (Wright et al., 2016).

Synthetic organo‐selenium pharmaceutical compounds, such as ebselen and ebsulphur, function as bifunctional small molecule chaperones for various mutant forms of SOD1 by forming covalent bonds with Cys111 (Capper et al., 2018). The SOD1 dimer interface groove can accommodate two ebselen molecules, binding to opposing Cys111 residues and expanding the hydrophobic SOD1 homodimer interface through aromatic π–π stacking of adjacent ligands. As a result, the affinity between monomers significantly increases, particularly for the SOD1 (A4V) mutant, shifting the monomer-dimer equilibrium towards the dimer (Capper et al., 2018). Ebsulphur exhibits similar binding properties. Importantly, ebselen does not interfere with the SOD1-CCS interaction (Capper et al., 2018), giving it a significant advantage over other Cys111-binding compounds, such as cisplatin. Ebselen is also known for its anti-inflammatory, antioxidant, and cytoprotective properties (Schewe, 1995; Saito et al., 1998; Yamaguchi et al., 1998; Wood-Allum et al., 2006). However, achieving target engagement and specificity remains challenging due to the abundance of free thiols and the large number of ebselen-binding proteins within cells (Chen et al., 2018). Thus, a medium-throughput screen using a differential scanning fluorescence assay to investigate the stabilization of SOD1 (A4V) mutants by ebselen derivatives was developed, aiming for improved SOD1 dimer stabilization and drug-like properties (Amporndanai et al., 2020; Chantadul et al., 2020). Potent candidates exhibited remarkable neuroprotective activities without observable toxicity in cells, while *in vivo* experiments with a selected compound demonstrated a significant delay in ALS disease onset in SOD1(G93A) mice, showcasing its potential therapeutic effect (Amporndanai et al., 2020).

#### Targeting the Trp32 residue

The sole tryptophan residue within SOD1 has been identified as a key factor in the misfolding of both mutant and wild-type proteins, through oxidative and prion-like aggregation mechanisms (Coelho et al., 2014; Pokrishevsky et al., 2018; Duval et al., 2019; Gill et al., 2019; Manjula et al., 2019; Crown et al., 2020). Trp32, residing on the solvent-exposed external layer of the protein, distanced from the Zn/Cu active site and the homodimer interface, is subject to oxidative post-translational modifications *in vivo* (Medinas et al., 2010; Figueroa et al., 2020). The bicarbonate-dependent peroxidase activity of SOD1 further enhances the oxidation rate of Trp32 *in vitro* (Coelho et al., 2014), while upon chronic ER stress conditions Trp32 oxidation is increased in cytosolic and mitochondrial extracts from transgenic WT SOD1 mice (Medinas et al., 2018). Site-directed mutagenesis studies have emphasized the pivotal role of Trp32 in aggregate formation as substitution of this residue in SOD1 (G93A) cultured motor neurons (Taylor et al., 2007) and in HEK cells expressing SOD1 (G127X) and SOD1 (G85R) (Grad et al., 2011) considerably reduced mutant cytotoxicity and aggregation propensity.

Prion-like propagation and *in vivo* conversion of WT SOD1 to a misfolded state linked to Trp32, has been identified following exposure to misfolded SOD1 mutant seed species. SOD1 (G127X) can convert WT SOD1 to the misfolded state in various cell lines (Grad et al., 2011) while SOD1 (G93A) induces WT SOD1 aggregation accelerating disease progression in mice (Bruijn et al., 1998; Deng et al., 2006; Wang et al., 2009; Ayers et al., 2016). Notably, human SOD1 mutants did not induce misfolding of murine SOD1 protein both *in vivo* (Wang et al., 2009; Audet et al., 2010) and in cells (Grad et al., 2011), due to a different codon for the residue at position 32 between humans and mice. This differentiation, from a tryptophan to a serine, has been associated with the observed aggregation resistance in mice. Intriguingly, the SOD1(W32F) variant significantly reduced the formation of high-molecular weight and disulfide-dependent SOD1 aggregates in NSC-34 cells (Medinas et al., 2018). Moreover, zebrafish embryos injected with SOD1(W32S) showed mitigated motor deficiency and motor neuron axonopathy compared to embryos injected with WT or ALS-related SOD1 variants (Duval et al., 2019), supporting the notion that reducing SOD1 self-interactions at Trp32 might be of therapeutic interest.

Recognizing the role of Trp32 oxidation in potentiating protein aggregation and cytotoxicity has spurred the development of small molecules targeting Trp32 in SOD1 (Ray et al., 2005; Antonyuk et al., 2010; Wright et al., 2013; Manjula et al., 2019). Pyrimidine-like molecules, such as 5-Fluorouridine (5-FUrd), 5-Fluorouracil (5-FU) and Telbivudine (a synthetic thymine analogue), have all demonstrated the ability to decrease SOD1 aggregation in cellular and animal models of ALS (Pokrishevsky et al., 2018; Duval et al., 2019; Rando et al., 2019). In particular, 5-FU administration in SOD1 (G93A) transgenic mice delayed disease onset, increased survival and ameliorated motor performance deficits (Rando et al., 2019). Additionally, telbivudine improved motor performance and reduced SOD1 toxicity in a SOD1-ALS zebrafish model (Duval et al., 2019). Both 5-FU and telbivudine are licensed in the United States and EU as chemotherapeutic and antiviral agents, respectively, and importantly, both compounds can also penetrate the BBB (Formica et al., 2006; Ahrens and Manno, 2014).

#### Targeting the electrostatic loop

The structure and activity of SOD1 depend on two important loops, the Zn-binding (residues 49-82) and the electrostatic loop (E-loop or loop VII, residues 121-142). A highly conserved cluster of charged residues situated on the surface of the electrostatic loop forms an active site channel, facilitating effective interaction with superoxide (Getzoff et al., 1992; Polticelli et al., 1995). NMR analysis of SOD1 folding and misfolding pathways identified thermal fluctuations within the electrostatic loop that mediate the formation of aberrant intermolecular interactions and the development of abnormal oligomers (Sekhar et al., 2015). Furthermore, structural and dynamic changes affecting this loop have been previously identified as a shared property of 13 familial ALS-related SOD1 variants (Molnar et al., 2009), while mutations dispersed throughout the SOD1 sequence that destabilize the electrostatic loop aberrantly expose the metal site of the protein to water (Souza et al., 2019). Thus, the binding of small molecules to the loop could alleviate the impact of mutations on the structure. Interestingly, the cyclic peptide HGG^4F^FQ (^4F^F = 4-fluorophenylalanine), selected by an epitope-specific high-throughput screen for binding to the peptide sequence of the electrostatic loop, can bind to both mature and metal-free SOD1 at the electrostatic loop, stabilize the native state and accelerate the WT and SOD1 (G93A) folding rates (Bunck et al., 2018).

#### Targeting intramolecular disulfide cross-linking

Short segments of SOD1 have a tendency to aggregate into oligomers and fibrils through the establishment of intramolecular hydrogen bonds and hydrophobic interactions (Ivanova et al., 2014; Sangwan et al., 2017). Additionally, SOD1 can create non-native oligomers in which monomers are connected through intermolecular disulfide bonds (Furukawa et al., 2006; Toichi et al., 2013). Several symptomatic transgenic ALS mouse models exhibit disulfide cross-linked SOD1 multimers detected in insoluble fractions derived from the animals’ spinal cords (Furukawa et al., 2006; Anzai et al., 2017). Importantly, such multimers are absent from transgenic mice expressing a C-terminally truncated SOD1 lacking Cys146, suggesting that this residue has a significant role in disulfide-linked multimerization *in vivo* (Furukawa et al., 2006). A screening of 640 FDA-approved drugs was conducted to evaluate their impact on abnormally cross-linked SOD1 oligomers derived from metal deficient apo-SOD1 (G37R)^S−S^ in an *in vitro* assay. The goal was to identify compounds that could inhibit the formation of insoluble S-S oligomers. Six compounds, namely, simvastatin, lovastatin, mevastatin, miltefosine, alfacalcidol and calcitriol were discovered to significantly suppress solution turbidity (Anzai et al., 2016). Despite this finding, treatment of an ALS mouse model expressing human SOD1 (G93A) with simvastatin resulted in accelerated disease progression and shortened survival (Su et al., 2016).

### Inhibiting self-assembly and the aggregation process

Apart from stabilizing the SOD1 dimer and promoting proper folding to prevent SOD1 aggregation, molecules which can interfere with the self-assembly, the nucleation, or the fibrillation process are of high therapeutic interest. A prime example is Methylene Blue (MB), a phenothiazine dye, which has shown considerable potential in modulating the aggregation of various amyloidogenic proteins (Gureev et al., 2022). The ability of MB to readily cross the blood-brain barrier has significantly increased interest in its use as a therapeutic agent for central nervous system disorders (Rojas et al., 2012). *In vitro* assays have demonstrated that MB inhibits both the spontaneous amyloid aggregation of SOD1 and the elongation of pre-existing fibrils (Malik et al., 2020; Musteikyte et al., 2020). However, MB treatment of SOD1 (G93A) mice had no effect on motor neuron loss, or on the aggregation or misfolding of SOD1 (Audet et al., 2012).

#### Natural products

Flavonoids are the most common phytochemicals, exerting pharmacological effects and possessing antioxidant properties (Nday et al., 2015), metal chelator abilities (Nday et al., 2015), anti-inflammatory effects (Lin L. et al., 2015) and neuroprotective properties (Prakash and Sudhandiran, 2015). Importantly, some flavonoids can cross the BBB, rendering them attractive drug candidates for CNS diseases, including AD, PD and ALS (de Andrade Teles et al., 2018; Maher, 2019). Certain flavonoids such as myricetin, lacmoid, EGCG and curcumin (Zhao et al., 2018) have demonstrated the ability to reduce apo-SOD1 aggregation in an *in vitro* assay by restraining the nucleation step and inhibiting fibril elongation (Malik et al., 2020). Despite evidence of a direct inhibitory role in SOD1 aggregation (Malik et al., 2020), myricetin may exert its beneficial effects through additional cellular mechanisms. For example, the observed decrease in intracellular accumulation of ubiquitin-positive SOD1 inclusions in myricetin-treated cells expressing SOD1 (G37R) may also be mediated by modulation of the endogenous levels of Hsp70 and the quality control E3 ubiquitin ligase E6‐AP (Joshi et al., 2019). EGCG is another flavonoid that has shown beneficial effects on onset delay, motor function, neuroprotection and extension of the lifespan of SOD1 (G93A) mice (Koh et al., 2006; Xu et al., 2006), combined with the suppression of microglial activation and inflammation (Xu et al., 2006). Finally, curcumin in an *in vitro* assay, reduced the fibril formation propensity of DTT-treated SOD1, favoring the formation of small amorphous aggregates which are not cytotoxic (Bhatia et al., 2015).

#### Molecular tweezers

Molecular tweezers are small, horseshoe-shaped artificial receptors that can form complexes with guest molecules, such as a nucleic acids, carbohydrates, amino acids or proteins, via non-covalent interactions (Mbarek et al., 2019). The molecular tweezer CLR01 selectively binds to positively charged lysine and arginine residues, interfering with the self-assembly process by disrupting hydrophobic and electrostatic interactions, thereby preventing the aggregation process in various amyloidogenic proteins (Sinha et al., 2011; Attar et al., 2012; Acharya et al., 2014; Ferreira et al., 2014; Herzog et al., 2015; Lopes et al., 2015; Zheng et al., 2015; Vopel et al., 2017; Mittal et al., 2018; Malishev et al., 2021). CLR01 also inhibits mutant SOD1 aggregation, as measured by ThT fluorescence assays, but leads to a marginal reduction in disease duration in the SOD1 (G93A) ALS mouse model despite reduced levels of misfolded SOD1 in the spinal cord (Malik et al., 2019). Recently, Lys61 and Lys92 have been identified as the preferential binding sites of CLR01 to SOD1, with the authors providing further evidence of CLR01’s ability to stabilize the monomer (Samanta et al., 2022).

#### Peptides

Peptides constitute a promising class of therapeutic molecules (Wang L. et al., 2022). Synthetic peptides from structure-based designs possess the ability to function as beta-sheet breakers of amyloid forming proteins (Sievers et al., 2011; Young et al., 2017), and their use in preventing aggregate formation has demonstrated promising outcomes in various disorders, including Alzheimer’s, prion, and polyglutamic diseases (Truex and Nowick, 2016; Alam et al., 2017; Goyal et al., 2017; Mukundan et al., 2017; Ryan et al., 2018). Peptide inhibitors, as potential drug candidates, combine several advantages such as high specificity, low toxicity and good tolerance (Hard and Lendel, 2012; Goyal et al., 2017). The peptide SE-12 (LSGDHCIIGRTLVVHEKADD) has been shown to bind SOD1 variants and to direct the aggregation of ALS-related SOD1 mutants from the typical amyloid to an amorphous aggregation pathway, which is presumably less toxic (Banerjee et al., 2016). Further studies were based on this peptide for the *in silico* design of tripeptides with inhibitory activity against SOD1 (A4V) aggregation, identifying CGH as the one with the greatest binding affinity among the tested sequences (Srinivasan and Rajasekaran, 2019). However, none of these peptides have been evaluated in cells or *in vivo.* In another approach, a peptide library comprising engineered variants of the hyperthermophilic variant of protein G (HTB1) was constructed and subsequently evaluated for specificity and affinity by a yeast display platform, identifying HTB1M as a selective inhibitor of SOD1 (G93A) and SOD1 (G85R) aggregation (Banerjee et al., 2017). Moreover, HTB1M prevented the accumulation of misfolded proteins in living cells and reduced the cytotoxicity of SOD1 (G93A) expressed in NSC-34 motor neuron–like cells. Importantly, the peptide library was designed using a computational algorithm for mapping protein surfaces predisposed to HTB1 intermolecular interactions. This conversion of an IgG-binding domain into a targeted SOD1 inhibitor showcases the potential usefulness of rational design for discovering peptide drug candidates. Finally, a bacterial platform was used to screen a combinatorial library of cyclic tetra-, penta- and hexapeptides for discovering rescuers of the misfolding and aggregation of the ALS-associated variant SOD1 (A4V). Among other hits, this high-throughput screen revealed that cyclic pentapeptides with the consensus cyclo-T (Φ1, S)SΦ2W motif, where Φ1 is one of the hydrophobic (Φ) amino acids A, W or F, while Φ2 is V, W or F, are efficient rescuers of SOD1 (A4V) aggregation (Matis et al., 2017).

## Molecules targeting TDP-43 misfolding and aggregation

Despite its central role in cellular health and disease, most strategies for removing pathological TDP-43 primarily focus on cellular clearance pathways (i.e., ubiquitin/proteasome system, autophagy) (Babazadeh et al., 2023) rather than directly targeting TDP-43 to inhibit its aggregation (Buratti et al., 2005), possibly due to the lack of typical druggable pockets in its structure (Francois-Moutal et al., 2019). Additionally, TDP-43 demonstrates a significant tendency to aggregate either during or shortly after the purification process (Wright et al., 2020; Yang et al., 2023), constraining the development of high-throughput assays for screening molecules inhibiting TDP-43 misfolding and aggregation. Notably, the design of stable monomeric TDP-43 variants (i.e., double mutant S333D/S342D) with the ability to be purified as stable monomers enabled the identification of small molecules capable of stabilizing the protein’s native state and preventing aggregation (Yang et al., 2023). Compounds with the ability to modulate the aggregation pathways and toxicity of amyloidogenic proteins such as MB and curcumin (Lo Cascio et al., 2021; Gureev et al., 2022) also prevent TDP-43 aggregation. MB reduces cytoplasmic TDP-43 inclusions in cells (Yamashita et al., 2009) and suppresses mutant TDP-43-mediated toxicity in *C. elegans* and zebrafish ALS (Vaccaro et al., 2012; Vaccaro et al., 2013) but was ineffective in reducing disease symptoms and cytoplasmic accumulation of TDP-43 in transgenic TDP-43 (G348C) mice (Audet et al., 2012). Similarly, the improved curcumin analogue monocarbonyl dimethoxy-curcumin C has been shown to inhibit the aggregation of mutant TDP-43 and to diminish oxidative stress in cells (Duan et al., 2014).

### Targeting the C-terminal domain and LLPS

The extended C-terminal disordered region includes a hydrophobic α-helix (residues 320–340) and dispersed aromatic residues that facilitate the LLPS of TDP-43, allowing its incorporation to stress granules (Li et al., 2018). A substantial body of evidence indicates the Q/N-rich segment (residues 341–366) as the pivotal region for amyloid formation (Johnson et al., 2009; Budini et al., 2012; Kametani et al., 2016), further supported by the recently determined cryo-EM structure of aggregated TDP-43 (Arseni et al., 2022). Inhibitors that block the aggregation of TDP-43 mediated by the Q/N-rich segment without interfering with the hydrophobic segment’s ability to drive association with stress granules could be an effective therapeutic approach. Polyglutamine binding peptide 1 (QBP1) is an octapeptide, designed to prevent polyglutamine amyloid formation (Nagai et al., 2000; Nagai et al., 2003; Takeuchi and Nagai, 2017). QBP1 exhibits a relatively broad specificity, capable of hindering amyloid formation by various amyloid-prone polypeptides, including huntingtin, the yeast Sup35 prion domain, α-synuclein, and the functional amyloid CPEB3. However, it does not affect certain amyloids like Aβ (Bauer et al., 2010; Hervas et al., 2012; Popiel et al., 2013; Hervas et al., 2016). Biochemical *in vitro* assays showed that QBP1 binds to the Q/N rich region of TDP-43 C-terminal domain and inhibits its aggregation (Mompean et al., 2019).

The small planar molecule AIM4 [4,5-bis acridine] is an acridine derivative, which has been predicted to bind amino acids 288-319 in the C-terminal domain of TDP-43 (Girdhar et al., 2020). AIM4 exhibited pronounced inhibitory effects on TDP-43 aggregation in both a yeast model expressing TDP-43-YFP and *in vitro*, using a recombinantly purified C-terminal fragment spanning amino acids 193 to 414 (Prasad et al., 2016). Recently, the amyloid fiber-detecting molecules, 4′-dianilino-1,1′-binaphthyl-5,5′-disulfonic acid (bis-ANS) and Congo Red, have been shown to modulate LLPS of TDP-43 (Babinchak et al., 2020). Importantly, these molecules allow TDP-43 LLPS at low concentrations while decondensing the liquid droplets at higher concentrations, indicating that they do not interfere with physiological phase transitions. Although bis-ANS and Congo red are toxic and therefore not suitable for drugs, their use as lead compounds could assist in the development of optimized molecules.

In an effort to design aggregation blockers, Liu and coworkers developed peptides based on sequence motifs of TDP-43. Through this approach, they discovered that two synthetic peptides from the C-terminal region effectively hindered the formation of TDP-43 protein aggregates in a concentration-dependent manner within HeLa cells overexpressing a mutant TDP-43. However, it's noteworthy that despite their ability to reduce aggregation, these peptides did not mitigate or prevent cell death (Liu et al., 2013). In another attempt, TDP-43-derived peptides based on two core aggregation-prone motifs of TDP-43 (Saini and Chauhan, 2011), 246-EDLIIKGISV-255 and 311-MNFGAFSINP-320, have been functionalized to enhance desired properties (Gao et al., 2019). These multi-functional peptides comprise three moieties: (a) a hydrophobic motif consisting of adamantane, (b) a TDP-43-derived peptide and (c) a Tat (RRRQRRKKRG) or Poly-D-Arginine (D-Arg) tag, each one conferring to the functionality of the compound. Adamantane mimics the unfolded state of proteins, and consequently, adamantane-tagged proteins are directed to the degradation machineries of the cell (Coll-Martinez et al., 2020). Moreover, adamantane increases the BBB permeability of conjugated molecules (Tsuzuki et al., 1994). Tat or (D-Arg)_8_ tags are used to increase cell-permeability of conjugated molecules (Szabo et al., 2022). While the peptide M311/P320 was not further examined due to low solubility, the peptide E246/V255 exhibited a potent capacity to trigger TDP-43 degradation within cells and to diminish TDP-43-induced cytotoxicity. Additionally, it was effective in lowering TDP-43 levels in a transgenic *Drosophila* model. Importantly, peptides lacking the adamantane moiety had no effect.

### Targeting the RRMs

TDP-43 localization to stress granules (SGs) is mediated by both its RRM1 and the C-terminal domain (Colombrita et al., 2009; Dewey et al., 2011). To discover small-molecule modulators influencing SG formation and decrease TDP-43 accumulation in induced pluripotent stem cell-derived motor neurons (iPSC-MN) from ALS patients, a cellular screen was conducted using a 50,000-member small molecule library (Fang et al., 2019). The screen involved HEK293T and neural precursor cells treated with NaAsO_2_ to induce SG formation. Approximately 100 hits were identified, and among them, mitoxantrone, quinacrine and pyrvinium planar compounds which act as nucleic acid intercalating molecules, showed reduced recruitment of TDP-43 to SGs. More recently, it has been reported that small molecules inhibiting the binding of TDP-43 to RNA could reduce neuronal toxicity (Francois-Moutal et al., 2019). Using an amplified luminescent proximity assay, the investigators observed that compound rTRD01 displaced (G4C2)4 RNA of the disease-linked *c9orf72* gene from TDP-43 and improved larval neuromuscular coordination of *Drosophila* overexpressing mutant TDP-43 (Francois-Moutal et al., 2019). Despite being targeted to an aggregation-mediating domain of the protein, it has not been examined whether rTRD01 reduces TDP-43 aggregation propensity.

In another study, through *in silico* docking of 50,000 compounds to the N-terminal domain of TDP-43, a small molecule (nTRD22) that binds specifically to the NTD domain has been identified. Notably, nTRD22 induced allosteric modulation of the RRM of TDP-43, leading to reduced RNA binding *in vitro* (Mollasalehi et al., 2020). Furthermore, exposure of primary motor neurons to nTRD22 resulted in a decrease in TDP-43 protein levels, akin to the effect observed with TDP-43 RNA binding-deficient mutants (Zacco et al., 2018; Flores et al., 2019), supporting the idea that nTRD22 disrupts the binding of TDP-43 to RNA. Interestingly, in a *Drosophila* ALS model, nTRD22 alleviated motor impairment (Mollasalehi et al., 2020). Additionally, several studies have demonstrated that nucleotides rich in UG/TG sequences can impede the aggregation of TDP-43 using *in vitro* biochemical assays (Huang et al., 2013; Sun et al., 2014; Rengifo-Gonzalez et al., 2021) but also in cells (Yang et al., 2023).

Auranofin [gold (1+)-3,4,5- triacetyloxy- 6- (acetyloxymethyl) oxane-2 - thiolate-triethyl-phosphanium] is an organic gold thiol compound currently used to treat rheumatoid arthritis due to its anti-inflammatory properties (Madeira et al., 2013; Madeira et al., 2014; Madeira et al., 2015). Auranofin has been shown to inhibit TDP-43 self-interaction in mouse N2a neuroblastoma cells without affecting endogenous levels of the protein and without conferring cellular toxicity (Oberstadt et al., 2018). This effect of auranofin was discovered in a cell-based screen of 1280 pharmacologically active compounds where TDP-43 self-interaction was monitored by establishing a NanoBit luciferase complementation assay in which the presence of auranofin enhances the protein’s partitioning in the soluble fraction (Oberstadt et al., 2018). Although its mechanism of action is not known, it has been proposed that auranofin modulates the cysteine residues of TDP-43 located in the RRMs, thus affecting its self-interaction and eventually aggregation (Oberstadt et al., 2018). Importantly, auranofin holds desirable pharmacokinetic properties as it is able to reach the CNS of mice and rats after oral administration (Madeira et al., 2013), rendering it an attractive drug candidate.

## Concluding remarks

The role of protein aggregation in the pathogenesis of ALS is critical, owing to its adverse effects on neuronal function and survival. Proteins such as SOD1 and TDP-43, when aggregated, can disrupt cellular processes, impair protein quality control mechanisms, and trigger toxicity, ultimately resulting in the degeneration of motor neurons, a hallmark of ALS. Present therapeutic strategies aim to mitigate these effects through various approaches, including the degradation of mRNA or the inhibition of its translation, the clearance of protein aggregates *via* antibodies, the disruption of protein-protein interactions that facilitate aggregation, the prevention of aggregate formation through vaccines, and the activation of protein clearance pathways. Nonetheless, an approach focused on directly targeting misfolded proteins might offer a more precise method for altering the course of the disease. Such specific interventions could provide greater accuracy in mitigating the detrimental impacts of certain protein aggregates. A better understanding of the aggregation mechanisms could lead to the development of molecules or screening techniques aimed at particular stages of the fibrillation process. For instance, targeting the dimerization of SOD1 at late folding stages may not yield effective *in vivo* results. Instead, compounds that facilitate metal binding or disulfide bond formation at earlier stages of SOD1 maturation could prove more beneficial therapeutically. Information on how misfolded and aggregated species confer toxicity *via* gains and losses of activity may also guide candidate drug decisions.

Despite the authorization of Tofersen, an antisense oligonucleotide targeting SOD1, there remains a critical need for additional therapeutic interventions to manage SOD1 aggregation. The specificity of Tofersen for SOD1-linked familial ALS, which accounts for only a fraction of ALS cases, underscores the necessity for a broader range of therapeutic strategies that can effectively reduce SOD1 aggregation. In this context, a strategy aiming at restoring SOD1’s conformation and eventually its function could be invaluable, serving as either a standalone or complementary therapeutic intervention. Furthermore, the ubiquity of TDP-43 in ALS pathology across the majority of cases positions it as a primary focus of ALS research. Efforts to mitigate TDP-43 aggregation are promising and could benefit a broad spectrum of ALS patients. However, challenges such as differentiating between the protein’s physiological and pathological states within cells, and the obstacles in crossing the blood-brain barrier with therapeutic agents, must be addressed. Significantly, evidence from cellular and animal studies has shown that identifying compounds capable of inhibiting misfolding and/or aggregation or promoting disaggregation may lead to disease-modifying effects (both *in vitro* and *in vivo*, as summarized in Table 1), yet none has proceeded to clinical trials. This situation highlights the urgent need for continued research and development in this area.

**TABLE 1:** A summary of compounds identified by their ability to inhibit aggregation of TDP-43 or SOD1.

Compound	Target protein	Mechanism of action	Stage of development (level of evidence)^a^	References
Auranofin	TDP-43	N/A	Preclinical (cell assays)	Madeira et al. (2013), Oberstadt et al. (2018)
PolyQ binding peptide 1	TDP-43	N/A	Preclinical (*in vitro*)	Mompean et al. (2019)
AIM4	TDP-43	N/A	Preclinical (*in vitro*)	Girdhar et al. (2020)
TDP-43-derived peptides	TDP-43	Trigger TDP-43 degradation	Preclinical (*in vivo*- *Drosophila*)	Gao et al. (2019)
nTRD22	TDP-43	Allosteric modulation of the RRM	Preclinical (*in vivo*- *Drosophila*)	Mollasalehi et al. (2020)
Curcumin	TDP-43	Modulator of aggregation	Preclinical (cell assays)	Duan et al. (2014)
	SOD1		Preclinical (*in vitro*)	Bhatia et al. (2015)
Methylene Blue	TDP-43	Modulator of aggregation	Preclinical (*in vitro*; no effect *in vivo*)	Yamashita et al. (2009)
	SOD1		Preclinical (*in vitro*; no effect *in vivo*)	Musteikyte et al. (2020)
Maleimide	SOD1	Crosslinking at Cys111	Preclinical (*in vitro*)	Auclair et al. (2010), Gavrin et al. (2012)
1,2-dithiane-1-oxide (Cyclic disulphides)	SOD1	Crosslinking at Cys111	Preclinical (cell assays)	Donnelly et al. (2018)
Cisplatin	SOD1	Binding at Cys111	Licensed^b^ (*in vitro*)	Banci et al. (2012)
Ebselen	SOD1	Binding at Cys111	Licensed^b^ (*in vivo*)	Capper et al. (2018)
Ebsulphur			Preclinical (*in vitro*)	
5-Fluorouridine	SOD1	Attenuation of solvent-exposed hydrophobic Trp32	Preclinical (*in vivo*- Zebrafish)	Pokrishevsky et al. (2018), Rando et al. (2019)
5-Fluorouracil			Licensed^b^ (*in vivo*)	
Telbivudine	SOD1	Attenuation of solvent-exposed hydrophobic Trp32	Licensed^b^ (*in vivo*- Zebrafish)	Duval et al. (2019)
HGGF^4−fluorophenylalanine^Q	SOD1	Binding to the electrostatic loop	Preclinical (*in vitro*)	Bunck et al. (2018)
Myricetin	SOD1	Modulator of aggregation	Preclinical (cell assays)	Malik et al. (2020)
EGCG	SOD1	Modulator of aggregation	Preclinical (*in vivo*)	Malik et al. (2020)
Molecular tweezer CLR01	SOD1	Inhibits self-interactions	Preclinical (*in vitro*; no effect *in vivo*)	Malik et al. (2019), Samanta et al. (2022)
Arylsulfanyl pyrazolone (ASP) & derivatives	SOD1	Modulator of aggregation	Preclinical (cell assays)	Benmohamed et al. (2011), Chen et al. (2011), Limpert et al. (2013)
Cyclo- hexane-1,3-dione (CHD) & derivatives	SOD1	N/A	Preclinical (cell assays)	Benmohamed et al. (2011), Zhang et al. (2012b), Gavrin et al. (2012), Limpert et al. (2013)
Pyrimidine-2,4,6-trione (PYT) & derivatives	SOD1	N/A	Preclinical (cell assays)	Benmohamed et al. (2011), Limpert et al. (2013), Xia et al. (2014)
Simvastatin	SOD1	Target intramolecular disulfide cross-linking	Licensed^b^ (*in vitro*; no effect *in vivo*)	Anzai et al. (2016)
Alfacalcidol	SOD1	Target intramolecular disulfide cross-linking	Licensed^b^ (*in vitro*)	Anzai et al. (2016)
Miltefosine	SOD1	Target intramolecular disulfide cross-linking	Licensed^b^ (*in vitro*)	Anzai et al. (2016)
LSGDHCIIGRTLVVHEKADD	SOD1	Inhibits self-interaction	Preclinical (*in vitro*)	Banerjee et al. (2016)
HTB1M	SOD1	Inhibits self-interaction	Preclinical (cell assays)	Banerjee et al. (2017)
Cyclo-T (Φ1,S)SΦ2W motif, where Φ1 is one of the hydrophobic (Φ) amino acids A, W or F, while Φ2 is V, W or F	SOD1	N/A	Preclinical (*in vitro*)	Matis et al. (2017)

^a^ This column specifies the current stage of drug development and the type of evidence supporting each molecule’s ability to inhibit the aggregation of the target protein.

b “Licensed” denotes drugs that are already authorized for indications other than ALS.

The high rate of failure in ALS clinical trials can be attributed to multiple pivotal factors. Historically, ALS has been treated as a uniform condition, overlooking its inherent heterogeneity. Future research must categorize ALS patients by specific genetic determinants and disease progression rates. Enhancing the use of biomarkers, refining preclinical models, improving trial design, and facilitating early intervention are crucial strategies to advance the development of ALS therapies. A holistic approach that integrates target-specific *in vitro* screening, validation using patient-derived iPSC neurons and glia, and mechanistic studies in model organisms such as zebrafish, fruit flies, and mice, can enhance the prediction of therapeutic effectiveness and its translation to clinical settings.
